# Effect of acute salinity stress on ion homeostasis, Na^+^/K^+^-ATPase and histological structure in sea cucumber *Apostichopus japonicus*

**DOI:** 10.1186/s40064-016-3620-4

**Published:** 2016-11-15

**Authors:** Chenfan Geng, Yi Tian, Yanpeng Shang, Liqiang Wang, Yanan Jiang, Yaqing Chang

**Affiliations:** Key Laboratory of Mariculture and Stock Enhancement in North China’s Sea, Ministry of Agriculture, Dalian Ocean University, Dalian, 116023 China

**Keywords:** Sea cucumbers, Salinity, Coelomic fluid, Osmoregulation, Ion concentration, Histological structure

## Abstract

**Background:**

Sea cucumbers (*Apostichopus japonicus*) are an imperiled fauna exposed to a variety of environmental condition such as salinity and studies are urgently needed to assess their effects to guide aquaculture efforts. The effects of acute salinity stress on coelomic fluid osmotic pressure, ion concentrations, the activity of Na^+^/K^+^-ATPase in respiratory trees and the histological variations were measured to evaluate the salinity tolerance of sea cucumbers.

**Results:**

Significant correlations in osmotic pressure were observed between coelomic fluid and ambient environmental salinity. In coelomic fluid, Na^+^ concentration was observed fluctuated during salinity 18 psu and the inflection point presented at the 6 h. The Na^+^/K^+^-ATPase activity in respiratory trees indicated the “U-shaped” fluctuant change and the change trend was opposite with the Na^+^ concentration. The ions (K^+^, Cl^−^) concentration decreased and showed the same tendency at salinity 40 psu with salinity 18 psu. The total coelomocytes counts and phagocytosis of coelomic fluid Na^+^/K^+^-ATPase activity indicated fluctuating changes under different salinity stress. Histological variation revealed a negative relation between decreasing salt concentration and tissue integrity. Tissue damages were significantly observed in intestines, muscles and tube feet under low salinity environment (18, 23 and 27 psu). The connective tissue in intestines of *A. japonicus* exposed to 18 and 23 psu damaged and partly separated from the mucosal epithelium. The significant variations occurred in tube feet, which presented the swelling in connective tissue and a fracture in longitudinal muscles under low salinity (18 psu). The morphological change of tube feet showed the shrinkage of connective tissue under high salinity (40 psu). The amount of infusoria in the respiratory trees decreased or even disappeared in salinity treatment groups (18 and 23 psu).

**Conclusion:**

The results inferred that osmoconformity and ionoregulation were seen in sea cucumbers, which contributed to understand the salinity regulatory mechanisms of *A. japonicus* under acute salinity stress.

## Background

The sea cucumber (*Apostichopus japonicus*) is a representative economic marine organism due to its potential as an edible delicacy as well as a traditional Chinese medicine, which widely distributed in coastal areas of tropical and temperate zone (Yan et al. 2013; Yu et al. 2014). An increasing demand to supply the global markets, has led to the large scale of cultivation of sea cucumbers in northern China and Japan (Liao 1997; Yokoyama 2013; Yu et al. 2014). Until 2014, the production of *A. japonicus* in China has reached 193,705 t with the farming area of 214,945 ha (DOF 2014). However, there are still some questions need to be solved in the sea cucumber breeding industry. Sea cucumber aquaculture ponds located mainly in the intertidal zone, so fluctuation of salinity in its natural habitat frequently occurred due to water exchange, evaporation and precipitation (Dong et al. 2008; Wang et al. 2014; Bai et al. 2015). Sharp salinity changes may also affect the feed intake and higher energy utilization for osmoregulation resulting in poor growth of sea cucumbers, and can even lead to discharge intestine and death (Fankboner 2002). Salinity had significant impacts on sea cucumbers, affecting their metabolism, growth and survival (Vidolin et al. 2002; Fankboner 2002). Understanding sea cucumber’s regulating mechanism against abiotic stressors may provide insights to the evolution of the stress response systems in holothurid invertebrates.

Previous researches thought that the sea cucumber was usually thought to be stenohaline because they lack an obvious osmoregulatory organ. The previous study of Binyon (1972) and Diehl (1986) indicated that most echinoderms cannot adjust their coelomic fluids osmotically, but there may be maintenance of some ionic concentrations slightly different from environmental sea water. The latest studies put forward that adult sea cucumbers can tolerate a broader salinity ranging from 20 to 39 psu (Yuan et al. 2006, 2010; Bai et al. 2015). At present, the optimal salinity for *A. japonicus* growth is reported 29–32 psu (Tian et al. 2015). Some results indicated that sea cucumbers exhibit hypo-osmotic regulation in low salinity media and show hyper-osmotic regulation in high salinity media (Binyon 1972; Diehl 1986). Vidolin et al. (2002) reported that the gray sea cucumber (*Holothuria grisea*) could temporally regulate the osmotic pressure of its coelomic fluids by possibly reducing the permeability of its body wall.

Recently, the effects of salinity on the morphological and biochemical features of target organs in echinoderm and marine animals have been shown (Brunelli and Tripepi 2005; Bernabò et al. 2008; Putranto et al. 2014; Xu et al. 2015). Some reports indicated that extracellular anisosmotic regulation, as one of osmotic and ionic regulation processes, was responsible for the maintenance of the osmolality and performed by the action epithelial enzymes like Na^+^/K^+^-ATPase, V-ATPase, HCO_3_^−^ ATPase, carbonic anhydrase (Ferire et al. 2008; Garcon et al. 2013). Several reports on the cellular and molecular responses of osmoregulatory enzymes have been published under acute and long term salinity stress conditions in *Scylla paramamosain*, black tiger shrimp, white shrimp, green crab, killifish, and hermit crab (Chung and Lin 2006; de la Vega et al. 2007; Gao et al. 2012; Henry et al. 2006; Scott et al. 2005; Rhodes-Ondi and Turner 2010). The study in oysters showed that ion channels play important roles in oysters under short- and long-term hypoosmotic stress (Zhang et al. 2016). The studies indicated that oysters, as successful colonizers of intertidal zones and estuaries, are remarkably resilient against harsh salinity fluctuations (Guo et al. 2015). These recent studies have significantly improved our understanding of the resilient against harsh salinity change in oysters.

The osmotic pressure of coelomic fluid, ion concentrations, Na^+^/K^+^-ATPase activity and histological structures were measured and performed after the sea cucumbers were exposed to different salinities stress. The present work was contributed to understand the salinity adaption mechanisms of sea cucumbers.

## Results

### Physiological indexes in *A. japonicus* exposed to different salinities

Through the acute salinity stress, all experimental sea cucumbers survived under all salinity treatment.

### Osmoregulatory capability

The alteration in coelomic fluid osmotic pressure of *A. japonicus* after exposure to different salinities was shown in Table 1. At a salinity of 32 psu 922.3 ± 1.2 m Osmol kg^−1^, the osmotic pressure of the coelomic fluid in the control group was 916.7 ± 1.7 m Osmol kg ^−1^. The mean osmotic pressure of ambient sea water maintained 519.6 m Osmol kg^−1^ under salinity 18 psu. While the osmotic pressure of sea cucumber in coelomic fluid decreased rapidly to 646.0 ± 3.1 m Osmol kg^−1^ within 1.5 h under low salinity stress (18 psu), and then stabilized after 6 h until the end of the experiment. The Osmotic pressure of the coelomic fluid rapidly increased from 916.7 ± 1.8 to 1025.0 ± 7.8 m Osmol kg^−1^ after 1.5 h when transferred to 40 psu. The mean osmotic pressure of salinity 40 psu sea water was 1178.8 m Osmol kg^−1^. This level showed significant difference after 3 h exposed to high salinity 40 psu, earlier than low salinity treatment (*P* < 0.05). When the salinity was maintained at 40 psu for 12 and 48 h, the osmotic pressures in coelomic fluid were 1182.0 ± 5.0 m Osmol kg^−1^ and 1183.7 ± 4.3 m Osmol kg^−1^, respectively, which showed no significant difference with the ambient water osmotic pressure (12 h, 1180.0 ± 0.6 m Osmol kg^−1^; 48 h, 1178.3 ± 1.2 m Osmol kg^−1^). The results indicated that the coelomic fluid osmolality of sea cucumbers adjust to the external osmolality, and temporarily below the sea water, then recovery balance with sea water in later 12 h in high salinity.

**Table 1:** Osmotic pressure of the coelomic fluid in *A. japonicus* and the rearing water during the period of experimental exposure at different salinities

Osmotic pressure/times (mOsmol kg^−1^)	CF (18 psu)	SW (18 psu)	CF control (32 psu)	SW control (32 psu)	CF (40 psu)	SW (40 psu)
1.5 h	646.0 ± 3.1^Aa^	518.7 ± 0.3^Ba^	916.7 ± 1.8	922.3 ± 1.2	1025.0 ± 7.8^Ca^	1175.3 ± 2.0 ^Da^
3 h	580.0 ± 2.9^Ab^	520.7 ± 0.3^Ba^	916.7 ± 1.8	922.3 ± 1.2	1136.3 ± 8.2^Cb^	1180.0 ± 0.6 ^Da^
6 h	567.3 ± 14.4^Abc^	518.7 ± 0.3^Ba^	916.7 ± 1.8	922.3 ± 1.2	1164.3 ± 2.0^Cc^	1179.3 ± 0.9 ^Da^
12 h	532.0 ± 0.6^Ac^	520.3 ± 0.3^Ba^	916.7 ± 1.8	922.3 ± 1.2	1182.0 ± 5.0^Cc^	1180.0 ± 0.6^Ca^
24 h	543.7 ± 1.9^Acd^	520.0 ± 0.6^Ba^	916.7 ± 1.8	922.3 ± 1.2	1159.0 ± 2.6^Cbc^	1179.7 ± 0.9 ^Da^
48 h	537.7 ± 1.3^Ac^	520.0 ± 0.6^Ba^	916.7 ± 1.8	922.3 ± 1.2	1183.7 ± 4.3^Cc^	1178.3 ± 1.2^Ca^

Data in the same column having different *lower case letters* indicate significant difference (*P* < 0.05) among different time periods and data in the same row having different *capital letters* indicate significant difference between coelomic fluid and sea water (n = 3). Values are mean ± SE (n = 3)

*CF* coelomic fluid, *SW* sea water

### Ion concentrations

In the low salinity treatments, the concentration of coelomic fluid sodium ions in *A. japonicus* fluctuated between 117.49 ± 7.19 and 195.09 ± 14.79 mmol L^−1^ and the peak presented at 1.5 h, then decreased at 6 h, thereafter raised with prolonging stress 12 h, then declined at 48 h. The concentrations of coelomic fluid sodium ions at 6 and 48 h were accordant to the control group, while the concentrations at other points were significantly higher than the control group. Changes in potassium ion concentrations in the coelomic fluid of *A. japonicus* were significantly decreased from normally 28.91 ± 0.25 to 3.71 ± 0.05 mmol L^−1^. Coelomic fluid potassium levels at 6 h significantly dropped from 22.75 ± 0.23 to 3.31 ± 0.10 mmol L^−1^, and there was a significant difference among the different sampling time periods (*P* < 0.05) except for 12 and 24 h. Chloride ion concentrations in coelomic fluid of *A. japonicus* were found to have the same trend as potassium ion concentrations, which decreased from 111.81 ± 0.24 to 76.27 ± 0.12 mmol L^−1^ after 48 h of transfer to 18 psu. There was a significant difference between the control group and those at the other times during the experimental period (*P* < 0.05) except for 3 and 6 h. All these results were shown in Table 2.

**Table 2:** Effects of low salinity (18 psu) on the variations of physiological indexes of *A. japonicus*

Times/indexes	Na^+^ (mmol L^−1^)	K^+^ (mmol L^−1^)	Cl^−^ (mmol L^−1^)	Total counts of coelomocytes (cells mL^−1^)	The phagocytosis of coelomocytes
Control (32 psu)	130.64 ± 1.59^a^	28.91 ± 0.25^a^	111.81 ± 0.24^a^	3.43 ± 0.17 × 10^6a^	0.087 ± 0.004^b^
1.5 h	195.09 ± 14.79^bcd^	26.80 ± 0.16^b^	108.55 ± 1.15^b^	1.42 ± 0.12 × 10^6d^	0.115 ± 0.013^a^
3 h	182.24 ± 12.53^bc^	25.26 ± 0.11^c^	99.63 ± 0.74^c^	2.04 ± 0.08 × 10^6b^	0.061 ± 0.002^c^
6 h	117.49 ± 7.19^a^	22.75 ± 0.23^d^	98.84 ± 0.76^c^	1.69 ± 0.12 × 10^6bcd^	0.063 ± 0.004^bc^
12 h	184.51 ± 7.19^bc^	3.31 ± 0.10^f^	85.09 ± 0.23^d^	1.51 ± 0.14 × 10^6cd^	0.062 ± 0.005^bc^
24 h	171.50 ± 14.23^b^	3.13 ± 0.06^f^	79.57 ± 0.61^e^	1.93 ± 0.25 × 10^6bc^	0.073 ± 0.012^c^
48 h	128.55 ± 14.22^a^	3.71 ± 0.05^e^	76.27 ± 0.12^f^	2.11 ± 0.10 × 10^6b^	0.096 ± 0.021^b^

Within rows, different superscript letters indicate significant differences (*P* < 0.05). Values are mean ± SE (n = 3)

Acclimation of *A. japonicus* from normal sea water (32 psu) to 40 psu affected some coelomic fluid parameters, as shown in Table 3. Sodium concentrations during the whole experiment were significantly higher in specimens at salinity 40 psu than the control group. These concentrations tended to increase as time goes by, and reached the maximum 251.19 ± 3.49 mmol L^−1^ at 48 h. Potassium ion concentrations in the coelomic fluid of sea cucumber decreased rapidly from 28.91 ± 0.25 to 9.43 ± 0.16 mmol L^−1^ when transferred to 40 psu for 1.5 h. Thereafter, these levels maintained stable lower than the control group, ranging from 8.25 ± 0.06 to 8.86 ± 0.09 mmol L^−1^. Chloride ion concentrations in coelomic fluid of *A. japonicus* were similar tendency as potassium ion concentrations. The chloride ion concentrations decreased significantly with ambient high salinity (40 psu) as compared to the control group (*P* < 0.05). There was no significant difference between the levels at 1.5 h and those at 3, 6, 12, and 24 h.

**Table 3:** Effects of high salinity (40 psu) on the variations of physiological indexes of *A. japonicus*

Times/indexes	Na^+^ (mmol L^−1^)	K^+^ (mmol L^−1^)	Cl^−^ (mmol L^−1^)	Total counts of coelomocytes (cells mL^−1^)	The phagocytosis of coelomocytes
Control (32 psu)	130.64 ± 1.59^a^	28.91 ± 0.25^a^	111.81 ± 0.24^a^	3.43 ± 0.17 × 10^6c^	0.087 ± 0.004^b^
1.5 h	187.10 ± 3.98^bc^	9.43 ± 0.16^b^	54.43 ± 0.19^c^	1.33 ± 0.03 × 10^7e^	0.067 ± 0.002^a^
3 h	200.08 ± 5.09^bcd^	8.51 ± 0.08 ^cd^	54.89 ± 0.36^c^	1.73 ± 0.13 × 10^6a^	0.065 ± 0.001^a^
6 h	219.72 ± 4.74^d^	8.25 ± 0.06^d^	55.83 ± 0.23^bc^	2.70 ± 0.14 × 10^6b^	0.070 ± 0.002^a^
12 h	194.54 ± 6.70^bcd^	8.86 ± 0.05^c^	55.34 ± 0.31^c^	5.37 ± 0.15 × 10^6d^	0.067 ± 0.001^a^
24 h	207.55 ± 12.21 ^cd^	8.86 ± 0.09^c^	55.31 ± 0.11^c^	5.04 ± 0.25 × 10^6d^	0.067 ± 0.001^a^
48 h	251.19 ± 3.49^e^	8.61 ± 0.01 ^cd^	56.89 ± 0.26^b^	4.73 ± 0.27 × 10^6d^	0.122 ± 0.004^c^

Within rows, different superscript letters indicate significant differences (*P* < 0.05). Values are mean ± SE (n = 3)

The effect of salinity on coelomocytes was assessed by determining the total counts of coelomocytes. The number of coelomocytes declined significantly at salinity 18 psu compared with control group (Table 2). The activity of phagocytosis in the coelomic fluid of *A. japonicus* was significantly affected by the low salinity 18 psu (Table 2). The phagocytosis of coelomcytes increased at 1.5 h and then decreased and eventually consistent with the control group. Total counts of coelomocytes decreased firstly at 1.5, 3, 6 h and increased subsequently at 12 and 24 h under salinity 40 psu (Table 3). Compared with the control group (32 psu), phagocytosis declined significantly in salinity 40 psu treatments and then increase to 0.122 ± 0.004 in the end of the experiment.

### The Na^+^/K^+^-ATPase activity of respiratory trees

**Fig. 1:**
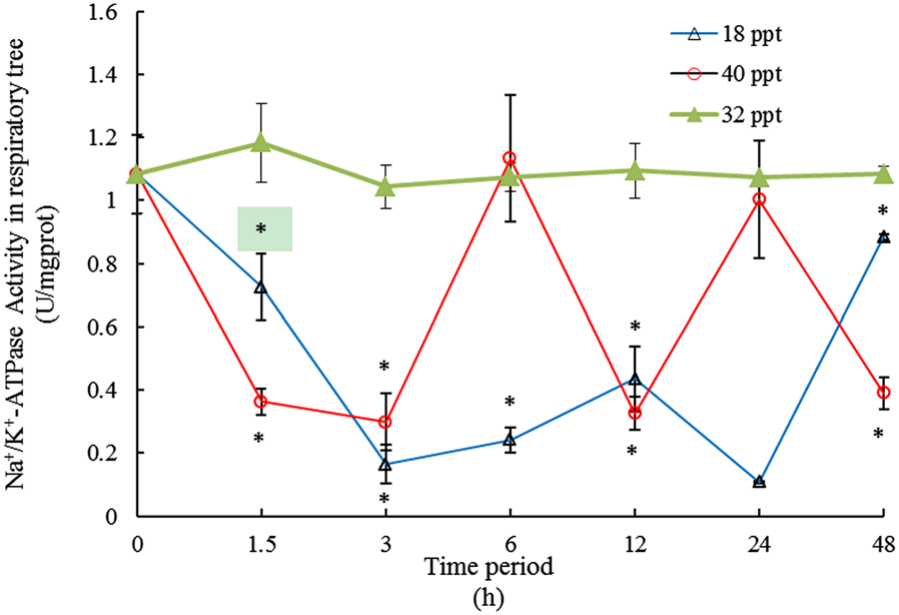
Na^+^/K^+^-ATPase activity in the respiratory trees of *A. japonicus* exposed to different salinities. An *asterisk* denotes significant difference between control and treatment groups (*P* < 0.05). Values are mean ± SE (n = 3)

The Na^+^/K^+^-ATPase activity (NKA) decreased compared to the control group, which presented fluctuation under salinity 18 and 40 psu in the study. The results for Na^+^/K^+^-ATPase activity of *A. japonicus* under different salinities are shown in Fig. 1. Under low salinity of 18 psu, the sampling values of Na^+^/K^+^-ATPase activity were significantly lower than that of the control group 1.08 ± 0.22 μmol ADP/mg protein/h. The Na^+^/K^+^-ATPase activity decreased and then increased, which presented the “U-shaped” fluctuant change during this period. Under salinity 40 psu, the Na^+^/K^+^-ATPase activities at 1.5, 3, 12 and 48 h were lower than that of control group, while the activities at 6 and 24 h were consistent with the control group. There were no significant differences among 1.5, 3, 12 and 48 h. Na^+^/K^+^-ATPase activity in respiratory trees went up significantly to (1.13 ± 0.20) μmol ADP/mg protein/h at 6 h and decreased dramatically thereafter and reached the levels observed at the control group. Na^+^/K^+^-ATPase activity of respiratory trees increased to 1.00 ± 0.19 μmol ADP/mg protein/h at 24 h and then decreased to 0.39 ± 0.05 μmol ADP/mg protein/h at 48 h. The K^+^ and Cl^−^ presented the “L-shaped” change under salinity 18 and 40 psu in the study, which had different inflection point with the Na^+^/K^+^-ATPase activity. The studies have shown a decrease in Na^+^/K^+^-ATPase activity at reduced salinities was accompanied by an increase in Na^+^ and a decrease in plasma Cl^−^.

### Histological structure of each tissue in sea cucumber exposed to different salinities

#### Variations of respiratory trees

**Fig. 2:**
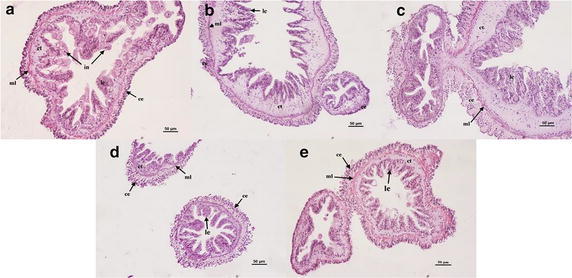
Light microscopy micrographs of the respiratory trees of *A. japonicus* (×20). **a** Control (32 psu); **b** salinity 18 psu; **c** salinity 23 psu; **d** salinity 27 psu; **e** salinity 40 psu. *ce* coelomic epithelium, *ml* muscular layer, *ct* connective tissue, *in* infusoria, *le* lining epithelium. *Scale bar* 50 μm

Eithelium of the cavity of the respiratory tree trunk in *A. japonicus* is composed of coelomic epithelium, muscular layer, connective tissue, lining epithelium and infusoria through outer to inner (Fig. 2a) (Spirina and Dolmatov 2001). Under a microscope in transverse section, the respiratory trees displayed a lumen filled with several parasitic ciliates in the control group (32 psu), in which other treatments cannot be found. Epidermis of mucosa was irregularly enclosed by the connective tissue (Fig. 2b). In this case, the mucosa remained intact, and the sub-epidermal connective tissue in respiratory trees showed swollen. There is no visible difference in histological changes of respiratory trees in 27 psu (Fig. 2d) and 40 psu salinity (Fig. 2e).

#### Variations of tube feet

**Fig. 3:**
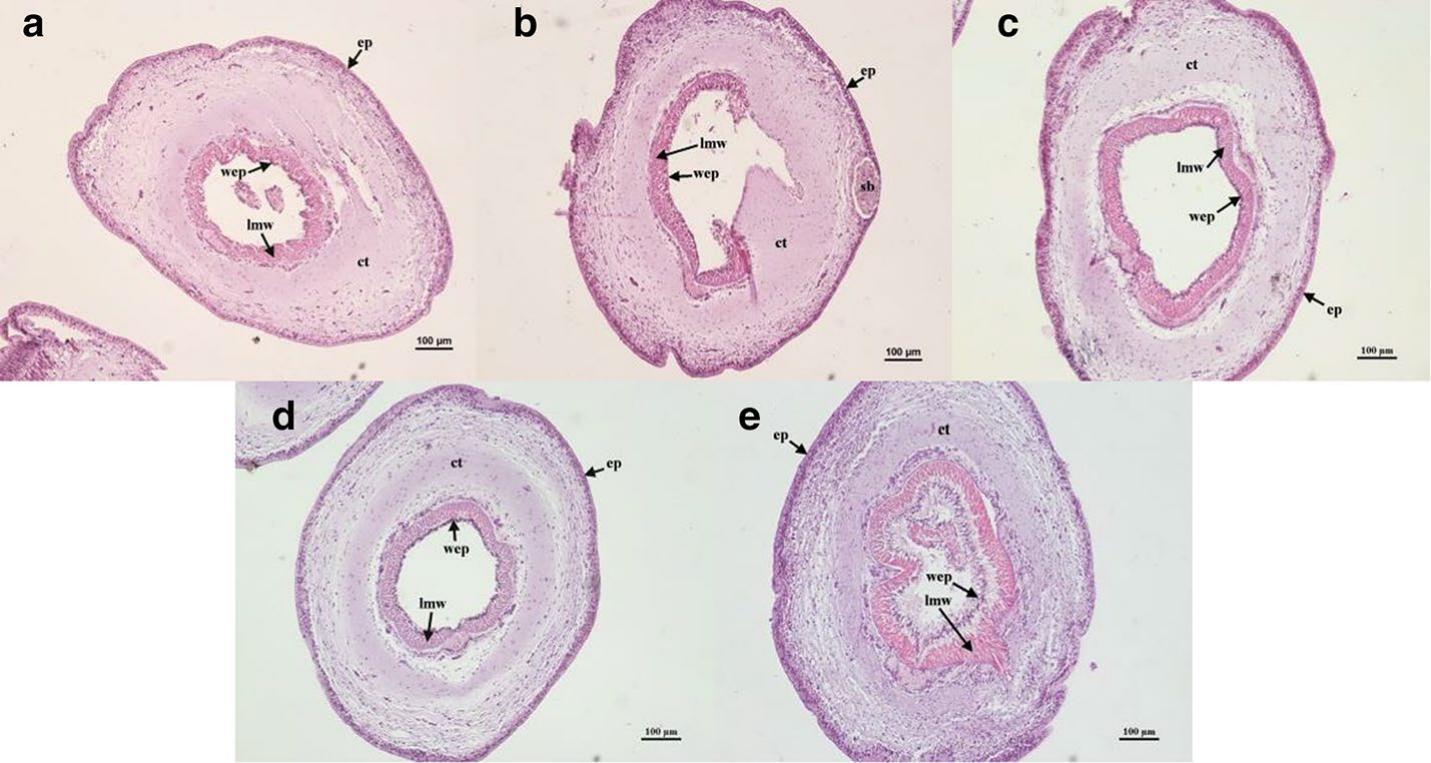
Light microscopy micrographs of tube-feet of *A. japonicus* (×10). **a** Control (32 psu); **b** salinity 18 psu; **c** salinity 23 psu; **d** salinity 27 psu; **e** salinity 40 psu. *ct* connective tissue, *ep* epidermis layer, *lmw* longitudinal muscle of water-vascular system, *sb* sensory band, *wep* epidermis of coelom. *Scale bar* 100 μm

The transverse section of a tube foot consists of an outer epidermal layer, a middle connective tissue, and an inner longitudinal muscle of water-vascular system (Fig. 3). In the control group (32 psu), the histological structure of tube feet were completely filled with a middle dense connective tissue, the longitudinal muscle of water-vascular naturally exists around the lumen of the tube feet (Fig. 3a). Exposure for low salinity can obviously examined the lacuna between the connective tissue and longitudinal muscle which caused by water absorption, swelling of the tube feet and resulted in the tissue damage (Fig. 3b–d). In the salinity 18 psu treatment, longitudinal muscle of water-vascular was destroyed and the tissue water absorption caused the loss of connective tissue. The loose connective tissue was presented in tube feet around the external longitudinal muscle of water-vascular in 23 psu (Fig. 3c). The tube feet contraction of *A. japonicus* exposed to salinity 40 were substantially found when compared with the tube feet of *A. japonicus* exposed to natural sea water. The shrinkage of water-vascular longitudinal muscle can be observed narrowed together at 40 psu (Fig. 3e). The tube-feet of *A. japonicus* submitted to both hypo- and hypersaline sea water displayed obviously tissue disruption.

#### Variations of intestine

**Fig. 4:**
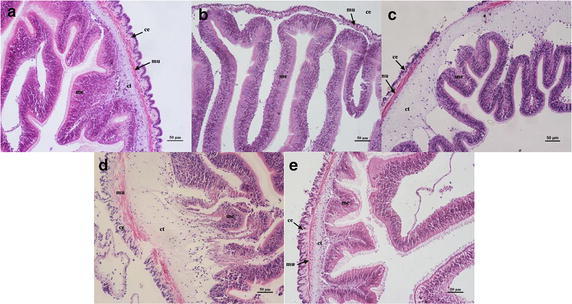
Light microscopy micrographs of intestine of *A. japonicus* (×20). **a** Control (32 psu); **b** salinity 18 psu; **c** salinity 23 psu; **d** salinity 27 psu; **e** salinity 40 psu. *ce* coelomic epidermis, *mu* muscular layer, *ct* connective tissue, *me* mucosal epithelium, *mu* muscular layer. *Scale bar* 50 μm

The intestine of *A. japonicus* has the similar tissue structure with the vertebrate intestine. The transverse sections can clearly observed the internal structure of the intestine. The intestine of *A. japnicus* consisted of a coelomic epithelial lining, an outer circular muscular layer, a longitudinal muscular layer, an inner connective tissue, and a pseudostratified mucosal epithelium (Fig. 4a). In low salinity stress, connective tissue diminished and separated from the pseudostratified mucosal epithelium, resulting in the cavities emerged between them. The connective tissue of *A. japonicus* which exposed to 18 psu suffered severely degenerated and almost disappeared (Fig. 4b). With low salinity 23 psu, morphological changes became more marked so that in some areas, coelomic epithelial lining and the connective tissue were partly separated from the mucosal epithelium. The coelomic epithelial injury can be obviously observed in 23 psu salinity treatment (Fig. 4c). In salinity 27 psu, mid-intestine displayed a damage coelomic epidermis and the broken connective tissue, with tissue apparently rupture (Fig. 4d). Changes of intestine structure showed no noticeable significant difference in 40 psu (Fig. 4e).

#### Variations of longitudinal muscles

**Fig. 5:**
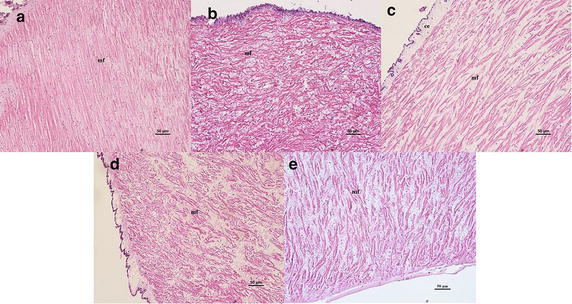
Light microscopy micrographs of muscle of *A. japonicus* (×20). **a** Control (32 psu), **b** salinity 18 psu, **c** salinity 23 psu, **d** salinity 27 psu, **e** salinity 40 psu. *ce* coelomic epithelium, *mf* muscle fibers. *Scale bar* 50 μm

Longitudinal muscle fibres of *A. japonicus* lined up regularly and tightly which exposed to the natural sea water salinity (32 psu) (Fig. 5a). After exposure to salinity 18, 23 and 27 psu, the longitudinal muscle of *A. japonicus* became irregular (Fig. 5b–d). The longitudinal muscle had a swollen appearance in low salinity stressed groups. Bundles of muscle fibers were presented irregularly twisted and were in disarray under salinity 18 psu treatment. After exposure to 40 psu, strong contraction of the longitudinal muscles caused the intuitional histological change (Fig. 5e).

## Discussion

### Effects of different salinities on the coelomic fluid osmotic pressure of *A. japonicus*

The osmotic pressure of coelomic fluid varied dramatically with salinity to acclimatize to ambient environment within the first 1.5 h and stabilized after 6 h in low salinity treatment. The results in osmotic pressure of coelomic fluid were similar with previous studies described (Dong et al. 2008; Meng et al. 2011; Wang et al. 2014), but not reached same osmotic pressure with ambient sea water during the experimental 48 h in low salinity 18 psu treatment. However, on exposure to high salinity (40 psu), the osmotic pressure of coelomic fluid increased rapidly in the initial 12 h until a new steady equilibrium status between the animal and the external medium was achieved. Then this level fluctuated slightly at 24 h, eventually reached osmotic balance with ambient sea water (40 psu) at 48 h. These results indicated that *A. japonicus* possesses a certain degree of salinity tolerance ability by ceolomic fluid adjustment and isosmotic intracellular regulation (Yuan et al. 2006, 2010; Bai et al. 2015). The studies in the oysters indicated oysters were remarkably resilient against harsh salinity fluctuations (Guo et al. 2015). These recent studies have significantly improved our understanding of the resilient against harsh salinity change in sea cucumbers. Previous studies showed that echinoderms become isosmotic with the ambient water by exchanging of water and ions in their coelomic fluid, and altering the concentrations of intracellular ions which play a vital role in metabolic processes and affect enzymes of intermediary metabolism (Yancey et al. 1982; Diehl 1986; Stickle and Diehl 1987; Meng et al. 2011). Vidolin et al. (2002) reported that the gray sea cucumber (*Holothuria grisea*) could temporally regulate the osmotic pressure of its coelomic fluids by possibly reducing the permeability of its body wall. These results indicated that adult *A. japonicus* could tolerate a wider range of salinity changes (18 and 40 psu) in short terms. It is suggested that *A. japonicus* can acclimatized gradually to an ambient salinity environment wider than the range of salinity affordable (20–39 psu) which had been published (Yuan et al. 2010; Zhang et al. 2012).

### Effects of different salinities on coelomic fluid ion concentrations and Na^+^-K^+^-ATPase activity of respiratory trees

The Na^+^/K^+^-ATPase activity (NKA) decreased compared to the control group, which presented the fluctuation change under salinity 18 and 40 psu in the study. The fluctuation of the activity of Na^+^/K^+^-ATPase indicated that the ion transport process belong to active transport which requires energy input to maintain ion homeostasis during this biological process. The external salinity environment stimulation created electrochemical gradient. The opening of ion channels permits the ions flow down dual gradient (Wood et al. 1998; Marshall and Grosell 2005; Melkikh and Seleznev 2012). This finding had also been observed in the Lebranch mullet, in which NKA presented the “U-shaped” model in the range of salinities test (Lisboa et al. 2015). Jensen et al. (1998) highlighted that this “U-shaped” pattern could represent an adaptive mechanism to improve the energy use since it allows fish to maintain a low gill NKA when facing a wide salinity gradient. Recent studies indicated that echinoderms could endure wide salinity fluctuation by isosmotic intracellecular regulation (Diehl 1986; Talbot and Lawrence 2002; Yuan et al. 2010) Osmoregulation can also be evaluated by measuring variation of Na^+^/K^+^-ATPase, with a variation trend likely indicating the operation of the ion transfer pump (Ostrowski et al. 2011). The study showed that the NKA activity reach consistent to the control group at 3, 6, 24 h and the concentrations of coelomic fluid sodium ions at 6 and 48 h were accordant to the optimum salinity under salinity 18 psu. Meanwhile under salinity 40 psu, the NKA activity reach consistent to the optimum salinity at 1.5, 3, 12, 48 h and the concentrations of coelomic fluid sodium ion presented “U-shape” pattern. Dong et al. (2008) reported that the osmotic pressure of the sea cucumber stabilized by 6 h after osmotic shock. The results inferred that the sea cucumber could tolerate wide salinity change and possessed certain adaptive mechanism by NKA activity, moreover specific detailed experiments need to formulate and verify the adaptive mechanism. The study indicated that concentration of coelomic fluid sodium ions indicated opposite tendency to Na^+^/K^+^-ATPase activity. Changes in Cl^−^ and K^+^ concentrations in the coelomic fluid of *A. japonicus* were significantly decreased contrast to control group, which was consistent to the activity of Na^+^/K^+^-ATPase activity. The Na^+^/K^+^-ATPase or Na pump extrude 3Na^+^ in exchange for 2K^+^ and thereby establish a strong cytosolic negative membrane potential and low intracellular Na^+^ concentrations, which play important role in for maintaining salt and water balance. So many researches indicated that Na^+^/K^+^-ATPase activity relates directly to Na^+^ and Cl^−^ fluxes across gills (Flik et al. 1997). Several studies have shown how a decrease in Na^+^/K^+^-ATPase activity at reduced salinities is accompanied by an increase in Na^+^ (Gaumet et al. 1995; Imsland et al. 2008) and a decrease in plasma Cl^−^ (Woo and Chung 1995; Foss et al. 2001; Fielder et al. 2007; Partridge and Lymbery 2008). This relation was found in the present study.

Isosmotic intracellular regulation involves coordinated changes in intracellular concentrations of both organic and inorganic coelomocytes not only to regulate cell volume but also to re-establish ionic balances for functional physiological processes (Diehl and Lawerence 1985). The respiratory tree is responsible for gas exchange and breathing metabolism (Zhao et al. 2014). Due to the specific respiratory organ of sea cucumbers, the activity of Na^+^-K^+^-ATPase in respiratory trees decreased when under low salinity of 18 psu at 1.5 h and significantly increased at 48 h. Unlike other kinds of marine animals whose respiratory rely on the gills, for instance, the amphibians, the crustacean and teleost (Purcell and Blockmans 2009; Huong et al. 2010; Seale et al. 2014; Rubino et al. 2015), the ability of osmoregulation of respiratory trees is poor than other higher animals.

### Histological changes in different tissues

Salinity stress has multiple impacts on marine organisms, including obvious damage on their morphological structures. Decreasing salinity had an overall effect on the Ark Shell (*Scapharca subcrenata*) including the epithelial layer necrosis in the gills as well as increased numbers of hemocytes, nuclear condensation, and cytoplasmic enlargement in the digestive glands (Shin et al. 2009). In addition, there was evidence that salinity events resulted in highly vacuolated hepatocytes in liver and even caused severe kidney damage of *Chalcalburnus chalcoides aralensis* (Wang et al. 2008). In the present study of *A. japonicus*, the histological structure damages were especially acute in tube feet, intestine and longitudinal muscles. Thus, we speculated these tissues might be more sensitive to salinity stress. The respiratory trees, as the distinct respiratory organ of *A. japonicus,* are responsible for gas exchange, osmotic regulation and room for excretion of metabolized production (Liu et al. 2005; Wang et al. 2009). Several parasitic ciliates congregated in the center antrum kept close to epidermis of mucosa in the respiratory trees when exposed to the normal rearing sea water. No parasitic ciliates were found in different salinity treatment groups, which may be caused by parasitic ciliates itself due to its poor tolerance ability of varied salinities. Gradient salinity processing may inactivate the respiratory metabolism of *A. japonicus.* The respiratory trees of *A. japonicus* possess similar function to the gills of aquatic animals. When compared to other species, the structural damage of the gills observed in *M. sintangense*, after it was exposed to the acute toxicity of cadmium at different low salinities (Putranto et al. 2014). Therefore, histological and ultrastructural change of gills can interfere with respiratory function and osmotic regulation (Bernabò et al. 2013). Tube feet, the unique mechano-sensory adhesive organs, belong to a part of the water vascular system of Echinoderms (Hennebert et al. 2008; Santos et al. 2013). The morphology of tube feet damages was conspicuous and easily distinguishable on the sections. This was being driven by sea cucumber which under inaptitude ambient salinity loose energy and possessed poor ability to adhere to rocks or the attachment plaques. Santos et al. (2013) reported that the connective tissue of sea urchin is the only tissue layer bearing the load. The mechanical properties of connective tissue give the tube feet an ideal balance of extensibility, strength and stiffness, which together produced a material with adequate toughness to absorb the impact of waves and currents, and thus to resist the environmental challenges of habitats in which sea urchins lived. In the present study, the severe histological changes of tube feet indicated the water salinity is one of the most important environmental factors affecting the movement and attachment of *A. japonicus*. The structural changes of intestine tissue of the present study were consistent with a previous study from Xu et al. (2015), which described that the structural changes in all tissue layers in intestine expect serosa and showed rapid and significant degradation under heat stress. The degradation in intestine tissue implied weak functions of digestion and absorption of nutrition under environmental stress, which would affect the growth. The similar study of Kim et al. (2013) showed that the loose of arrangement of connective tissue and decrease of mucous cells in dermal layer of integumentary system of *A. japonicus* were observed when exposed to below the salinity of 20 psu. Integumentary system of the sea cucumber exposed a salinity of 40 psu mainly observed nucleus hypertrophy of epithelial cells, increase of mucous cells and tight arrangement of connective tissue in dermal layer. The gastrointestinal tract is particularly responsive to stressors, which can cause a variety of changes including alteration of normal protective microflora and decreased integrity of the intestinal epithelium (Longo and Díaz 2015; Carvalho et al. 2015). Histological variations revealed a negative relation between decreasing salt concentration and tissue integrity. All the tissue damages would deteriorate with the increasing stress time.

## Conclusions

Salinity stress could impact on ion homeostasis, Na^+^/K^+^-ATPase and histological structure of sea cucumber at different salinities. The osmotic pressure, ion homeostasis and Na^+^/K^+^-ATPase activity changed with the changing of the ambient salinity accordingly. The studies had shown a decrease in Na^+^/K^+^-ATPase activity at reduced salinities is accompanied by an increase in Na^+^ and a decrease in plasma Cl^−^. Salinity stress has multiple impacts on sea cucumber organisms, including obvious damage and degradation on their morphological structures. The results inferred that the sea cucumber could tolerate wide salinity change and possessed certain adaptive mechanism by NKA activity, moreover specific detailed experiments need to formulate and verify the adaptive mechanism.

## Methods

### Experimental design

Based on previous studies of sea cucumbers’ adaptation salinity, two extreme salinities were designed as the aimed salinities (18 and 40 psu) for observing physiological indexes variations (Fu et al. 2014). The sea cucumbers living in natural sea water were included as control group (32 psu). The number of sea cucumbers for each treatment was 30. After being transferred to the pre-prepared seawater at 18 and 40 psu, three randomly selected sea cucumbers in each group were sampled at 0, 1.5, 3, 6, 12, 24 and 48 h, respectively. Three repeats were applied and above samples were used to measured physiological indexes. Meanwhile 30 sea cucumbers which used in histological structure analysis were divided into five groups according to different salinity conditions (18, 23, 27, 32 and 40 psu). Each tissue was dissected from three randomly selected sea cucumbers of each salinity group which acclimatized the ambient environment for at least 48 h.

### Source and acclimatization of sea cucumbers

The adult *A. japonicus*, with average body wet weight of 25 ± 0.33 g (mean ± SE, n = 3), were collected from Wafangdian Aquatic Farm (salinity 32 psu), Dalian, P.R. China. The sea cucumbers were acclimated in three aquariums (90 × 75 × 60 cm) in Dalian Ocean University, Key Laboratory of Mari-culture and Stock Enhancement in North China’s Sea, Ministry of Agriculture for 2 weeks. The tap water was dechlorinated and inflated before use in experiments by letting it stand for at least 24 h to allow the chlorine to evaporate from the water (O'Beirn et al. 1998). Low salinity level was achieved by reducing the salinity of sea-water 2 psu per day by adding freshwater. High salinity level added crude sea salt to reach the aimed salinity. Control animals were cultured at a salinity of 32 psu. One-half or two-thirds of the rearing water was changed daily and remain stable with aimed salinity during the acclimation period. The sediment (feces and uneaten food) were removed by siphon method. During the experiment, sea cucumbers were fed once per day, at the rate of 3–5% wet weight with a laboratory made formulated diet (mix up a solution of proportional parts of *Sargassum thunbergii*, fish meal and sea mud). The salinity and other water parameters were measured using YSI multi-parameter water quality monitor (YSI, USA). Throughout acclimation, temperature, pH and dissolved oxygen were measured as 24–25 °C, 7.92–8.29, 3.76–5.56 mg L^−1^, respectively.

### Osmotic pressure

About 500 μL coelomic fluid was extracted using 1.0 mL disposable syringe and to make sure that all the fluid was from the coelom. Be carefully, the tip of the needle was used pierced the body wall until it reached the coelomic cavity at a small angle. Approximately, 50 μL of coelomic fluid was extracted, and transferred to 60 μl dry and cleaning testing tube. The osmotic pressures of coelomic fluid (CF) and rearing water (SW) were measured using a Gonotec 030 Cryoscopic Osmometer (Gonotec instruments, German). Osmotic pressure is expressed as mOsmol kg^−1^.

### Ion concentrations and enzyme assay

The ion concentrations of Na^+^, K^+^, Cl^−^ in coelomic fluid and total protein and Na^+^/K^+^-ATPase activity of respiratory trees were measured using the corresponding biochemistry assay kit which provided by Nanjing Jiancheng Bioengineering Institute (Nanjing, China).

1. The determination of sodium ions

The Na^+^ contributed to the homogeneously turbidity with 6-KSb(OH)_4_ in the existence of dispersants and removal interference agents. Then use enzyme-labelling instrument to measure its OD value in 630 nm wavelength. The calculation formula is shown as follows.

$$ [{\text{Na}}^{ + } ]({\text{mmol/L}}) = \frac{\text{D} - \text{C}}{\text{N} - \text{C}} \times {\text{S(140 mmol/L)}} \times {\text{X}} $$

where D is the OD values of determining hole, C is the OD values of control hole, N is the OD values of norm hole, S is standard sample concentration, X is the diluted multiples of pre-experimental sample.

2. The determination of potassium ions

Under alkaline medium, the K^+^ in serum samples and NA-TPB react together and produce uniform and stable suspension through the reaction of protein precipitation. Then use enzyme-labelling instrument to measure its OD value in 450 nm wavelength. The calculation formula is similar with the determination of sodium except the standard sample concentration. The standard sample concentration of potassium ion is 0.4 mmol L^−1^.

3. The determination of chloride ions

Because of Hg(SCN)_2_ with Cl^−^ to form a non-ferrous complex, the color depth was proportional to the concentration of Cl^−^. Then use enzyme-labelling instrument to measure its OD value in 480 nm wavelength with similar calculation formula except the difference in standard sample concentration which is 20 mmol L^−1^.

4. The determination of the activity of Na^+^/K^+^-ATPase

Through ultrasonic disruption, the tissue homogenate were obtained from respiratory trees of each salinity which was restored in −80 °C refrigerator. One enzyme activity unit means 1 μmol inorganic phosphorus which produced by the procedure of ATP was decomposed into ADP per hour in per ml ceolomic fluid protein. The equation is shown as follows.

$$ \begin{aligned} {\text{The activity of NKA (U/mg prot) = }}\frac{\text{D} - \text{C}}{\text{N} - \text{C}} \times 6 \times 7.8/{\text{P}} \hfill \\ {\text{Total protein concentration (g/L) = }}\frac{\text{D} - \text{C}}{\text{N} - \text{C}} \times {\text{S (0}} . 5 6 3 {\text{ g/L)}} \hfill \\ \end{aligned} $$

where D is the OD values of determining hole, C is the OD values of control hole, N is the OD values of norm hole, S is standard sample concentration, P is the concentration of total protein.

### Total counts and the phagocytosis of coelomocytes

The number of coelomocytes was counted on a hemacytometer under a microscope and the number of coelomocytes per mL of coelomic fluid was calculated for each specimen. Phagocytosis was detected according to Zhao et al. (2012), with minor modifications, using neutral red yeast as the test particle. Three replicates of 50 μL sea cucumber coelomic fluid were transferred into a 96-well tissue culture plate, incubated for 1 h at 4 °C and added 100 μL of sea water normal saline (0.02 M HEPES, 0.4 M NaCl, 0.1 M MgSO_4_, 0.01 M KCl, 0.01 M CaCl_2_) to regulate pH to 7.4. And then washed twice, added 50 μL neutral red yeast suspension to incubate for 30 min at 20 °C and terminated by adding 100 μL BFC. Redundant yeast suspension solution was washed by saline, after that added 100 μL cell lysis buffer (acetic acid:ethanol = 1:1). The results were recorded with Epoch biotech microplate reader at 550 nm. The absorbance of 10^6^ cells indicates the capability of coelomocytes phagocytosing neutral red.

### Histological structure

The tissue samples collected from each salinity group were fixed by Bouin liquid for 24 h, then rinsed the fixed tissue using 70% alcohol, dehydrated by ethanol in different concentrations (70, 85, 90, 95 and 100%). Tissue samples were clarification in xylene, embedded in paraffin wax with a mean fusion point of 56 °C, sectioned at 6 μm thickness on a microtome (Leica RM 2235, German), and stained by hematoxylin and eosin. The cell and tissue changes involved with salinity stress were examined with a microscope (Nikon Eclipse 50i, Japan). The important histological characteristics were photographed using a Nikon Digital Sight microscopy camera (Japan).

### Statistical analyses

Results were expressed as mean ± SE. Statistical analyses were performed with SPSS version 13.0 (SPSS Inc, Chicago, Illinois, USA). Statistical differences between groups and different treatment time were analyzed by One-way analysis of variance. Duncan’s multiple range tests were used to compare two data sets with 95% confidence intervals. A value of *P* < 0.05 was considered significant.

## Abbreviations

NKA: Na^+^/K^+^-ATPase activity
ce: coelomic epithelium
ml: muscular layer
ct: connective tissue
in: infusoria
le: lining epithelium
ep: epidermis layer
lmw: longitudinal muscle of water-vascular system
sb: sensory band
wep: epidermis of coelom
mu: muscular layer
me: mucosal epithelium
mf: muscle fibers
